# Novel Quantitative Trait Loci for Grain Cadmium Content Identified in Hard White Spring Wheat

**DOI:** 10.3389/fpls.2021.756741

**Published:** 2021-12-02

**Authors:** Ling Qiao, Justin Wheeler, Rui Wang, Kyle Isham, Natalie Klassen, Weidong Zhao, Meng Su, Junli Zhang, Jun Zheng, Jianli Chen

**Affiliations:** ^1^Institute of Wheat Research, State Key Laboratory of Sustainable Dryland Agriculture (in preparation), Shanxi Agricultural University, Linfen, China; ^2^Department of Plant Sciences, University of Idaho, Aberdeen, ID, United States; ^3^Department of Plant Sciences, University of California, Davis, Davis, CA, United States

**Keywords:** cadmium, grain, spring wheat, doubled haploid population, quantitative trait locus

## Abstract

Cadmium (Cd) is a heavy metal that can cause a variety of adverse effects on human health, including cancer. Wheat comprises approximately 20% of the human diet worldwide; therefore, reducing the concentrations of Cd in wheat grain will have significant impacts on the intake of Cd in food products. The tests for measuring the Cd content in grain are costly, and the content is affected significantly by soil pH. To facilitate breeding for low Cd content, this study sought to identify quantitative trait loci (QTL) and associated molecular markers that can be used in molecular breeding. One spring wheat population of 181 doubled haploid lines (DHLs), which was derived from a cross between two hard white spring wheat cultivars “UI Platinum” (UIP) and “LCS Star” (LCS), was assessed for the Cd content in grain in multiple field trials in Southeast Idaho, United States. Three major QTL regions, namely, *QCd.uia2-5B*, *QCd.uia2-7B*, and *QCd.uia2-7D*, were identified on chromosomes 5B, 7B, and 7D, respectively. All genes in these three QTL regions were identified from the NCBI database. However, three genes related to the uptake and transport of Cd were used in the candidate gene analysis. The sequences of *TraesCS5B02G388000* (*TaHMA3*) in the *QCd.uia2-5B* region and *TraesCS7B02G320900* (*TaHMA2*) and *TraesCS7B02G322900* (*TaMSRMK3*) in the *QCd.uia2-7B* region were compared between UIP and LCS. *TaHMA2* on 7B is proposed for the first time as a candidate gene for grain Cd content in wheat. A KASP marker associated with this gene was developed and it will be further validated in near-isogenic lines *via* a gene-editing system in future studies.

## Introduction

Wheat (*Triticum aestivum* L.) is a critically important food crop, providing 20% of the calories consumed by the population worldwide. Due to environmental pollution and climate change, wheat faces many challenges, including biological and abiotic stress (Bowne et al., 2012; Wegulo, 2012; Guzmán et al., 2016). Recently, heavy metal stress has attracted increased attention (Rizwan et al., 2016). Compared to other heavy metals, cadmium (Cd) is more toxic, has higher bioavailability, and is more easily accumulated in crops. Therefore, Cd pollution is an important risk factor for the environment and human health (Shi et al., 2019).

Cd causes serious problems for both crop production and the human diet (Wagner and Donald, 1993; Prasad, 1995). A long-term intake of food with high levels of Cd causes human health risks, including itai-itai disease, cardiovascular disease, cancer, chronic kidney disease, and bone disease. The maximum allowable Cd concentration in wheat grain is 0.2 mg kg^–1^, but only 30 μg kg^–1^ is the maximum allowable amount in baby food products (FAO/WHO, 2010). Two methods can be used to prevent Cd from entering the human food chain. The first method is to decrease plant availability by changing the form of Cd in soil. Soil acidification alters the form of Cd present in soil, increases the presence of Cd^2+^ and the bioavailability of Cd, and ultimately leads to increased Cd absorption and accumulation by plants (Naidu et al., 1994; Zeng et al., 2011). The second method is to breed crop cultivars that take up and accumulate less Cd. Studying the genetic basis of Cd uptake and transport in crops will contribute to the breeding approach.

The mechanism of Cd absorption and transport by plants has been described in rice. There are two mechanisms for Cd to enter plant root cells. First, Cd can enter plants *via* the same mechanisms used for the absorption of essential mineral elements, such as Mn, Zn, and Fe (Nakanishi et al., 2006; Lu et al., 2009; Takahashi et al., 2011a,b; Ishimaru et al., 2012; Sasaki et al., 2012; Song et al., 2014). Second, Cd can enter plants *via* chelation with small molecules such as plant-chelating peptides and enter the root cells in the form of Cd phytochelatins and other binding states (Clemens, 2006). *OsNRAMP5* is a transshipment protein gene involved in the absorption of external Mn^2+^, Cd^2+^, and Fe^3+^ by rice root cells (Ishimaru et al., 2012; Yang et al., 2014). The ability of xylem-mediated Cd transfer from roots to aerial parts determines the Cd content in rice stems and grains (Clemens and Ma, 2016). *OsHMA3* can transport Cd^2+^ into vacuoles to isolate and reduce Cd transport to the aboveground parts, thereby reducing the Cd toxicity (Ueno et al., 2010; Miyadate et al., 2011; Sasaki et al., 2014; Lu et al., 2019). Cd is transported from roots to the aboveground parts and then from phloem to various tissues and organs. Uraguchi et al. (2011) isolated the Cd transport protein gene *OsLCT1*, which was mainly expressed in leaves and stem nodes at the reproductive stage of rice. The *CAL1* gene played an important regulatory role in the process of Cd distribution in the aboveground parts of rice (Luo et al., 2018). In durum wheat, a single dominant gene, *Cdu1-B* located on chromosome 5B, was associated with low Cd concentration (Penner et al., 1995; Clarke et al., 1997; Knox et al., 2009; Abuhammad et al., 2016; Oladzad et al., 2018; Salsman et al., 2018), accounting for more than 80% of variation in the accumulation of Cd in grain (Wiebe et al., 2010; Harris and Taylor, 2013). Maccaferri et al. (2019) discovered a metal transporter gene (*TdHMA3-B1*) on chromosome 5BL, with a non-functional variant causing high accumulation of Cd in grain.

Genetic studies of Cd in common wheat lag that of rice and durum wheat due to the large genome size. Two quantitative trait loci (QTL) for the accumulation of Cd were identified on wheat chromosomes 4A and 5D, explaining up to 17% of phenotypic variation (Ci et al., 2012). Ban et al. (2020) identified additional two QTL for low Cd content in grain on chromosomes 4BS and 6BL. Using genome-wide association scans, Guttieri et al. (2015) identified Cd-associated single-nucleotide polymorphisms (SNPs) on 5AL in a region homologous to *Cdu1* locus on 5BL in durum wheat. Zhang et al. (2020) found three *TaHMA3* genes (i.e., *TaHMA3-A1*, *TaHMA3-B1*, and *TaHMA3-D1*) in common wheat, all of which encode transporters located in the vacuolar membrane. The absolute expression level of these genes was very low in all three wheat cultivars compared with that of *OsHMA3* in rice, especially in the roots.

Genetic research on the uptake and accumulation of Cd in crops is generally lacking. The QTL identification is the foundation of gene cloning and molecular marker-assisted breeding. Therefore, research to discover QTL for Cd content and associated molecular markers under different soil pH and Cd conditions will have both theoretical and practical values.

The objectives of this study were to identify QTL and to analyze potential candidate genes for grain Cd content in spring wheat in relation to the genes controlling the grain Cd content in durum and rice.

## Materials and Methods

### Plant Materials

This study used 181 doubled haploid lines (DHLs) which were developed using a wheat × maize hybridization system (Laurie and Bennett, 1986) through the services of Heartland Plant Innovation in Kansas, United States. The DHLs were derived from the F_1_ generation of a cross between two high yielding hard white spring wheat cultivars, namely, UI Platinum (UIP) and LCS Star (LCS). UIP was developed by the University of Idaho Agricultural Experiment Station and released in 2014 (Chen et al., 2016). LCS was developed and released by Limagrain Cereal Seeds. Both parents have a semi-dwarfing allele at the *Rht-B1* locus and similar plant height but have alternative alleles for the two major photoperiod response genes. UIP has the photoperiod insensitive alleles at loci for both *PPD-B1b* and *PPD-D1b*, while LCS has the sensitive alleles. As a result, UIP flowers earlier than LCS when grown under short-day conditions.

This study also used 127 spring wheat cultivars and elite lines to validate QTL identified in the DHLs. These lines were from multiple wheat breeding programs in the Pacific Northwest of the United States and the International Maize and Wheat Improvement Center (CIMMYT) in Mexico, as described by Wang et al. (2017).

### Field Evaluation

The parents and DHLs were planted and assessed in four-field trials, with two dryland trials in Soda Springs (SS), ID (42°43′ N, 111°35′ W, altitude 1,760 m) in 2017 and 2018 (17SS and 18SS), and two irrigated trials in Ashton (AS), ID (44°4′ N, 111°23′ W, altitude 1,603 m) in 2017 and 2018 (17AS and 18AS). The 127 spring wheat cultivars and elite lines were planted in the same field as DHLs in SS in 2017. The soil in both the locations had pH < 6, but AS had lower pH and lower Cd content than that in SS (Table 1).

**TABLE 1:** Content of four metals and pH in 0–30 cm soil in two irrigated and two non-irrigated field trials.

**Trial^ϕ^**	**Soil type**	**Irrigation**	**pH**	**Cd mg kg^–1^**	**Zn mg kg^–1^**	**Mn mg kg^–1^**	**Fe mg kg^–1^**
17SS	Silt Loam	None	5.3–6.0	0.57	1.34	22.82	54.52
18SS	Silt Loam	None	4.6–6.0	0.62	1.55	45.95	108.28
17AS	Silt Loam	Some	4.4–5.4	0.18	1.74	60.87	186.9
18AS	Silt Loam	Some	4.7–5.4	0.25	1.52	35.47	319.25

*^ϕ^17, 2017; 18, 2018; SS, Soda Springs; AS, Ashton.*

The two 2018 trials used seven row plots with 3.0 m in length, 1.5 m in width, and 0.25 m between the rows. The DH and parental lines were arranged in a randomized complete block design with two replications. Because of limited seed, the two 2017 trials had one replicate of four row plots with 1.5 m in width, 1.5 m in length, and 0.5 m between the rows. Field management in both SS and AS used common field practices, and the plots were managed by cooperating growers.

### Sample Collection and Preparation for Elemental Analysis

Composite core soil samples were taken from each location to establish a baseline profile for N, P, K, Zn, Fe, Cu, Mn, S, Cd, Cr, Ni, Pb, soil type, organic matter, pH, and salinity. Soil was sampled by splitting the field into two parts based on the environmental layout of the field (i.e., slopes and dips in the field). Several core samples for each part were drawn at 15 and 30 cm. Samples were submitted as 0–15 and 15–30 cm to determine the depth profile of the elements in the soil. Full elemental analysis for macronutrients and micronutrients, including Cd levels, was conducted at the Utah State University analytical lab.^1^

Plots were harvested using a Wintersteiger Classic small plot combine (2002 Wintersteiger Elite, Wintersteiger Seedmech) equipped with a Harvest Master weighing system (HM-400, Juniper Systems, United States). From each plot, 300 g samples of grain were milled using a Perten 3100 Laboratory Mill (2012 Perten Instruments, United States). For each line, a 15 g subsample of milled whole grain flour was sent to the University of Idaho Analytical Sciences Laboratory for elemental analysis.

### Cadmium Analysis

Milled grain samples were digested in 30% nitric acid and Cd content measured by using inductively coupled plasma mass spectrometer collision/reaction (ICP-MScx). Samples were prepared by using the SMM.57.070.05 protocol maintained by the University of Idaho Analytical Sciences Laboratory. The concentrations of Cd in the milled samples were determined using an Agilent 7800 inductively coupled plasma mass spectrometer (ICP-MS) (Agilent 7800 ICP-MScx, United States).

### Data Analysis

The content of Cd in each grain sample from all trials was used in the subsequent analysis. The best linear unbiased prediction (BLUP) and the broad-sense heritability (*H*^2^) were calculated from data sets across years and locations by using SAS V8.0 (SAS Institute, Cary, NC, United States) (Smith et al., 1998). The rate of decrease (DR) of Cd content in grain across the range of soil pH from the trials was calculated as follows: (AS - SS)/AS × 100, where AS is the mean Cd content in grain from all AS trails and SS is the mean for SS. The Spearman’s correlations of Cd content across four trials were calculated, and BLUP data were derived from multiple trials.

### Genetic Map and Quantitative Trait Loci Analysis

A genetic map of the mapping population was generated with 14,236 polymorphic SNPs from the wheat Illumina 90k SNP assay, representing all 21 hexaploid wheat chromosomes (Isham et al., 2021). The 7DS linkage map also included additional seven KASP markers published by Isham et al. (2021). All linkage details were used in QTL analysis in this study.

QTL analysis was conducted using individual and BLUP data sets for grain Cd content by using the composite interval mapping (CIM) method in JMP Genomics 9.0.^2^ Significant QTL were determined with the expectation maximization algorithm at a threshold of 2.5 [logarithm of the odds (LOD) ≥ 2.5] (Lin et al., 1996). The names for QTL followed the International Rules of Genetic Nomenclature.^3^ The software output provided a proportion of phenotypic variance (*R*^2^) and the additive effects for each marker. The source of the allelic effect of the parent UIP or LCS was indicated by negative or positive estimates of the additive effects, respectively. The LOD threshold of 2.5 was set for entry and retention in the model. Epistatic analysis was performed with the IciMappingVer.4.1 EPI module (LOD = 5, step = 1 cM, PIN = 0.0001).

To determine the physical positions for identified QTL regions, a BLAST search^4^ was performed to align the QTL-associated peak and flanking SNP marker sequences with the reference wheat genome assembly constructed in the cv. Chinese Spring (CS) sequence (RefSeq v1.0, the International Wheat Genome Consortium).

### Candidate Gene Analysis and Validation for the Major Quantitative Trait Loci Identified

Genes within the target region were identified using the genome browser (JBrowse) on the triticeae multiomics website (Triticeae Multi-omics).^5^ The sequences of common wheat genes were retrieved based on the intervals of major QTL identified from https://urgi.versailles.inra.fr/download/iwgsc/IWGSC_RefSeq_Annotations/v1.0/ (Zheng et al., 2019). The sequences were used to perform a BLAST search against the genome sequence databases of rice^6^ and durum wheat^7^ to identify orthologous gene pairs. The collinearity of these genes was analyzed using MCscan (Python version).^8^ Functional annotation and enrichment analysis of genes in segments were carried out in the Gene Ontology (GO) database using the R package cluster Profiler.

The candidate genes that were related to the uptake and transport of Cd were used in comparative analysis between the two parental lines using the resequencing data generated by the program of the corresponding author (Chen, personal communication). Gene-specific markers were designed based on the sequence differences between UIP and LCS. The markers were genotyped in the original mapping population and in 127 diverse lines in the validation panel. The contribution of the candidate genes to the Cd content in grain was validated based on the association analysis between marker data and the grain Cd content, and the allelic effect of the candidate gene was analyzed with a *t*-test in SAS V8.0.

## Results

### Phenotypic Variation and Correlations of Cadmium in Four Environments

The Cd content in LCS was generally higher than that in UIP, except in 18SS. Based on the BLUP value, the Cd content in grain was 0.108 mg kg^–1^ for LCS, 0.099 mg kg^–1^ for UIP, and 0.083–0.126 mg kg^–1^ for the DHLs (Table 2). The Cd content of the parents and DHLs were lower than the maximum level of grain Cd proposed by FAO/WHO (0.200 mg kg^–1^) (FAO/WHO, 2010). The estimated *H*^2^ for the Cd content was 0.68, which was high (*H*^2^ > 0.50), indicating that it was affected more by genetic vs. environmental factors (Table 2). However, the Spearman’s correlations ranged from 0.313 to 0.414 across different trials, indicating that the Cd content of the DHLs is affected by environment (Table 3). The Cd content of the DHLs showed continuous variation, suggesting multigene genetic control.

**TABLE 2:** Phenotypic performance and distribution of Cd content (mg kg^–1^) in parents and the doubled haploid lines in four-field trials.

**Trial**	**Parents**		**DHLs**				
	**LCS Star**	**UI Platinum**	**MAX**	**MIN**	**Mean**	**SD^ϕ^**	***H* ^2^**
18SS	0.076	0.079	0.138	0.029	0.079	0.019	0.68
18AS	0.114*	0.087*	0.140	0.061	0.096	0.016	
17SS	0.112	0.095	0.187	0.058	0.106	0.028	
17AS	0.146	0.132	0.182	0.065	0.121	0.021	
BLUP	0.108	0.099	0.127	0.082	0.101	0.010	

*^ϕ^SD, standard deviation; H^2^, broad-sense heritability; BLUP, best linear unbiased prediction.*

*Significant at P < 0.05.

**TABLE 3:** Correlation coefficients for Cd content among four trials.

**Trial**	**18SS**	**18AS**	**17AS**
18AS	0.396**		
17AS	0.336**	0.414**	
17SS	0.313**	0.316**	0.324**

**Significant at P < 0.01.

### Quantitative Trait Loci for Cadmium Content in Grain

**FIGURE 1:**
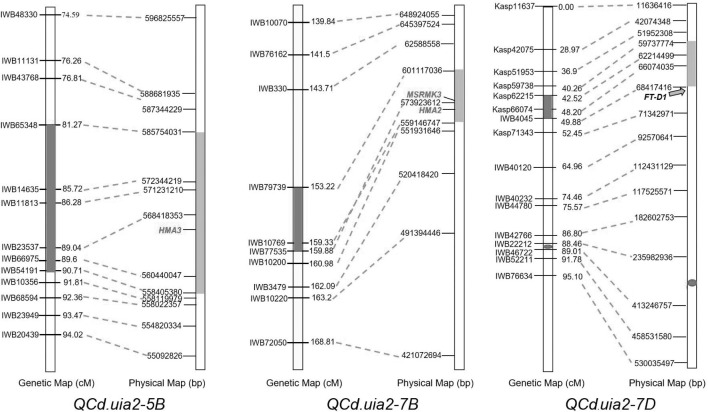
Comparative genetic linkage and physical maps of *QCd.uia2-5B*, *QCd.uia2-7B*, and *QCd.uia2-7D*.

A total of 10 QTL explaining 6.37–15.34% of the phenotypic variance were detected (Table 4). Three major QTL regions, namely, *QCd.uia2-5B*, *QCd.uia2-7B*, and *QCd.uia2-7D*, were detected in more than three environments (Table 4 and Figure 1). LCS had alleles for higher Cd content at *QCd.uia2-5B* and *QCd.uia2-7B*, and UIP had an allele for higher Cd content at *QCd.uia2-7D* (Table 4). *QCd.uia2-5B* was detected in three data sets, namely, 18SS, 18AS, and BLUP, explaining 7.2–11.1% of grain Cd content. It was physically mapped in a 558.41–585.75 Mb interval on 5BL. *QCd.uia2-5B* is near *Cdu1-B* on chromosome 5BL (Penner et al., 1995; Clarke et al., 1997; Abuhammad et al., 2016; Oladzad et al., 2018; Salsman et al., 2018). *QCd.uia2-7B* was detected in three data sets, namely, 18AS, 17AS, and BLUP, explaining 7.2–10.6% of grain Cd content. It was physically mapped in a 559.14–601.17 Mb interval on 7BL. *QCd.uia2-7D* was detected in four data sets, namely, 17AS, 18AS, 18SS, and BLUP, explaining 7.63–12.29% of grain Cd content. It was physically mapped in a 59.74–68.42 Mb interval on 7DS. No Cd content-related QTL have been reported earlier in these two intervals; therefore, *QCd.uia2-7B* and *QCd.uia2-7D* are likely novel. The other seven minor QTL were only detected in one to two data sets, explaining 6–15% of phenotypic variation (Table 4).

**TABLE 4:** Major quantitative trait loci (QTL) for Cd content (mg kg^–1^) in grain detected in the DH population.

**QTL**	**Trial**	**Chr**	**Peak marker**	**Marker interval**	**Genetic distance (cM)**	**Physical distance (Mb)**	**LOD**	***R*^2^ (%)**	**Add**
*QCd.uia2-5B*	18SS	5B	*IWB66975*	*IWB65348-IWB54191*	81.27–90.71	558.40–585.75	4.64	11.14	129.04
	18AS	5B	*IWB66975*	*IWB65348-IWB54191*	81.27–90.71	558.40–585.75	4.54	10.92	100.77
	BLUP	5B	*IWB66975*	*IWB65348-IWB54191*	81.27–90.71	558.40–585.75	2.95	7.23	48.21
*QCd.uia2-7B*	18AS	7B	*IWB10769*	*IWB77535-IWB79739*	153.22–159.88	559.14–601.17	4.42	10.63	110.23
	17AS	7B	*IWB10769*	*IWB77535-IWB79739*	153.22–159.88	559.14–601.17	2.92	7.15	106.63
	BLUP	7B	*IWB10769*	*IWB77535-IWB79739*	153.22–159.88	559.14–601.17	3.41	8.30	50.62
*QCd.uia2-7D*	18SS	7D	*IWB4045*	*Kasp59738-Kasp71343*	40.26–49.88	59.74–68.42	3.58	8.70	−110.02
	18AS	7D	*IWB4045*	*Kasp59738-Kasp71343*	40.26–52.45	59.74–71.34	5.10	12.29	−123.48
	17AS	7D	*IWB4045*	*Kasp62215-IWB4045*	40.26–49.88	59.74–68.42	4.69	11.26	−138.03
	BLUP	7D	*IWB4045*	*Kasp62215-IWB4045*	40.26–49.88	59.74–68.42	5.25	7.63	−67.05
*QCd.uia2-2A.1*	18AS	2A	*IWB30196*	*IWB30196-IWB34575*	75.09–102.37	16.08–36.63	3.72	9.04	−88.42
*QCd.uia2-2A.2*	17AS	2A	*IWB6858*	*IWB42945-IWB8574*	27.28–65.03	36.93–50.51	6.55	15.34	177.33
*QCd.uia2-2D*	17AS	2D	*IWB53594*	*IWB53594-IWB64250*	22.44–27.43	481.60–577.15	3.72	9.04	131.17
*QCd.uia2-4B*	17SS	4B	*IWB10640*	*IWB41888-IWB10640*	87.47–95.21	644.47–649.82	2.65	6.51	136.93
	BLUP	4B	*IWB10640*	*IWB41888-IWB10640*	87.47–95.21	644.47–649.82	2.59	6.37	42.48
*QCd.uia2-4D*	17AS	4D	*IWB10207*	*IWB19222-IWB10053*	26.42–58.9	366.27–499.10	4.46	10.73	−138.50
*QCd.uia2-5D*	18AS	5D	*IWB79949*	*IWB79949-IWB34503*	16.91–28.72	32.70–221.01	2.97	7.27	83.33
*QCd.uia2-6A*	18AS	6A	*IWB45465*	*IWB11269-IWB45465*	41.47–44.81	568.50–646.63	3.17	7.76	78.77

### Effects of Major Quantitative Trait Loci, *QCd.uia2-5B*, *QCd.uia2-7B*, and *QCd.uia2-7D* on the Cadmium Content

**FIGURE 2:**
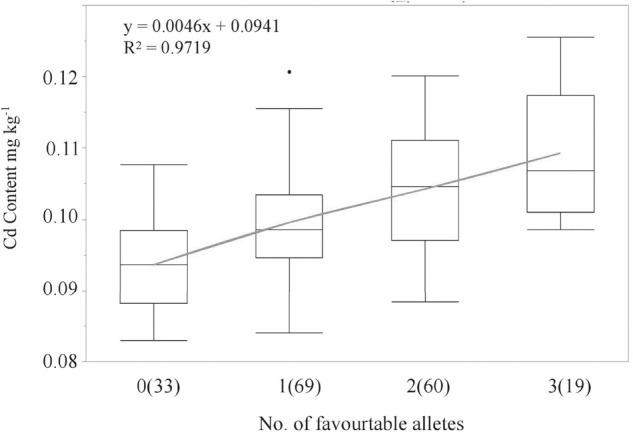
Linear regressions between the number of high Cd alleles (number of lines) and BLUP Cd content in the DH population. Numbers of lines carrying the corresponding number of favorable alleles are shown in brackets. X and Y in the equation represent the number of high Cd alleles and BLUP Cd content, respectively.

LCS contributed the Cd-increasing alleles for both *QCd.uia2-5B* and *QCd.uia2-7B* and UIP contributed the Cd-increasing allele for *QCd.uia2-7D*, and these three QTL regions have additive effects toward increasing Cd content in grain (Table 4 and Figure 1). There was no epistatic effect observed for the three major QTL regions (Supplementary Table 1). The average Cd content increased as the number of alleles increased (Table 5 and Figure 2). The DHLs with low Cd alleles at all the three QTL regions had 0.0161 mg kg^–1^ less Cd content compared to those with contrasting alleles. The combination of negative alleles from *QCd.uia2-5B*, *QCd.uia2-7B*, and *QCd.uia2-7D* had the largest effect on the Cd content.

**TABLE 5:** Additive effects of the QTL on 5B, 7B, and 7D for Cd content (mg kg^–1^) in grain across sites in the UI Platinum × LCS Star-derived double haploid population.

***QCd.uia2-5B***	***QCd. uia2-7B***	***QCd.uia2-7D***	**Sample size**	**Cd content (mg kg^–1^)**	**Difference^c^**
+^a^	+	+	19	0.1090 ± 0.0090e^b^	0
−	+	+	19	0.1064 ± 0.0088de	−0.0026
+	−	+	13	0.1043 ± 0.0100cde	−0.0047
+	+	−	28	0.1032 ± 0.0085bcd	−0.0058
−	−	+	15	0.0979 ± 0.0086bc	−0.0092
+	−	−	32	0.0998 ± 0.0096bc	−0.0089
−	+	−	22	0.0968 ± 0.0069ab	−0.0121
−	−	−	33	0.0929 ± 0.0079a	−0.0161

*^*a*^Plus and minus represent lines with and without the positive alleles of the target quantitative trait loci (QTL) based on the flanking markers and the corresponding QTL.*

*^*b*^All pair means were compared using the Tukey–Kramer HSD method. Values followed by the same lowercase letter are not significantly different at P = 0.05.*

*^*c*^Differences calculated using the entries with the three positive alleles minus the entries with three negative (increasing) alleles.*

### Candidate Gene Analysis of the Three Major Quantitative Trait Loci

*QCd.uia2-5B* was physically mapped in a 558.41–585.75 Mb interval on 5BL, and 293 genes were found in this interval in CS. Nine of these genes were involved in metal ion transport according to gene functional annotations in the GO public database (Table 6). The functional annotation of *TraesCS5B02G388000* was for the transport of Zn and Cd. The corresponding gene *TraesCS5B02G388000* in wheat was *TRITD5Bv1G197380* (*TdHMA3*) in durum wheat and *Os07g0232900* (*OsHMA3*) in rice. Therefore, *TraesCS5B02G388000* was named *TaHMA3* in wheat.

**TABLE 6:** Candidate genes significantly associated with Cd and either metal uptake or transport in the three major QTL regions identified in this study.

**Chr**	**Gene_ID**	**Gene annotation**	**Rice_gene_ID**	**Durum wheat_gene_ID**
5BL	*TraesCS5B02G386300*	Magnesium ion transmembrane transport	*Os03g0742400*	*TRITD5Bv1G196890*
	*TraesCS5B02G388000*	Cadmium ion and zinc ion transport	*Os07g0232900*	*TRITD5Bv1G197380*
	*TraesCS5B02G392600*	Metal cluster binding	*Os03g0748700*	*TRITD5Bv1G199360*
	*TraesCS5B02G395800*	Transition metal ion transport		
	*TraesCS5B02G396400*	Cellular metal ion homeostasis		
	*TraesCS5B02G397500*	Cellular metal ion homeostasis	*Os03g0755100*	*TRITD5Bv1G200950*
	*TraesCS5B02G397600*	Cellular metal ion homeostasis	*Os03g0755100*	*TRITD5Bv1G200960*
	*TraesCS5B02G400100*	Calcium ion transmembrane transport	*Os03g0758300*	*TRITD5Bv1G201760*
	*TraesCS5B02G402100*	Calcium ion transport	*Os03g0759600*	
7BL	*TraesCS7B02G318800*	Sodium ion transmembrane transport	*Os06g0701600*	
	*TraesCS7B02G319100*	Iron ion binding		
	*TraesCS7B02G320100*	Cadmium ion and zinc ion transport		
	*TraesCS7B02G320900*	Cadmium ion and zinc ion transport	*Os06g0700700*	*TRITD7Bv1G176040*
	*TraesCS7B02G321200*	Zinc ion and iron ion transmembrane transport		
	*TraesCS7B02G321400*	Cellular response to iron ion starvation		
	*TraesCS7B02G322900*	MAP kinase activity	*Os06g0699400*	
	*TraesCS7B02G323600*	RNA polymerase II transcription regulatory region sequence-specific DNA binding	*Os06g0698900*	*TRITD7Bv1G177350*
	*TraesCS7B02G324500*	Transmembrane receptor protein serine/threonine kinase activity		*TRITD7Bv1G178210*
	*TraesCS7B02G325300*	Transmembrane receptor protein serine/threonine kinase activity		*TRITD7Bv1G178420*
	*TraesCS7B02G333200*	Transmembrane receptor protein serine/threonine kinase activity	*Os06g0693200*	*TRITD7Bv1G182340*
	*TraesCS7B02G337700*	Cellular transition metal ion homeostasis		*TRITD7Bv1G184110*
	*TraesCS7B02G342500*	Negative regulation of transmembrane receptor protein serine/threonine kinase signaling pathway	*Os06g0687500*	*TRITD7Bv1G186350*
	*TraesCS7B02G342200*	Transcription regulatory region sequence-specific DNA binding		*TRITD7Bv1G186230*
7DS	*TraesCS7D02G100200*	Calcium ion transmembrane transport		.

The *QCd.uia2-7B* was physically mapped in a 559.14–601.17 Mb interval on 7BL and 307 genes were found in this interval in CS. Out of these genes, 12 were involved in the transmembrane transport of metal ions, such as Zn, Fe, and Cd (Table 6). *TraesCS7B02G320900* is homologous to *OsHMA2* (*Os06g0700700*) and *TraesCS7B02G322900* is homologous to *OsMSRMK3* (*Os06g0699400*) in rice. *TraesCS7B02G320900* and *TraesCS7B02G322900* were named *TaHMA2* and *TaMSRMK3*, respectively.

The interval of *QCd.uia2-7D* has 128 genes in CS. Only *TraesCS7D02G100200* participates in the transmembrane transport of Ca and other divalent cations (Table 6).

### DNA Sequencing Analysis and Protein Structure Prediction

**FIGURE 3:**
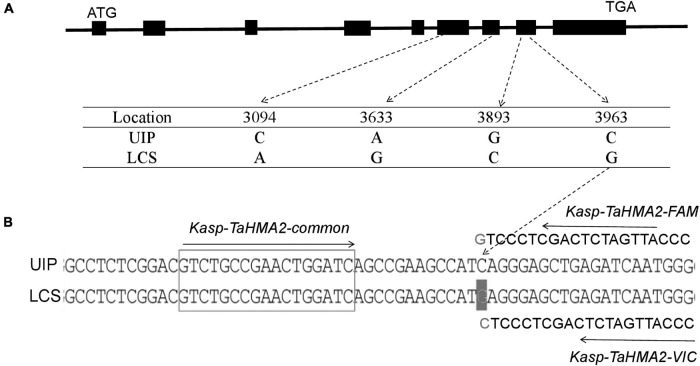
Sequence comparison of *TaHMA2* in two parents and KASP marker associated with the candidate gene. **(A)** Schematic diagram of nucleotide polymorphism for *TaHMA2*. The polymorphic site and relative positions are indicated on the genomic sequence of *TaHMA2*. Exons are indicated by black boxes, flanking regions and introns are indicated by solid black lines. **(B)** A KASP marker was designed using the nucleotide polymorphism of *TaHMA2*.

We analyzed the coding and promoter regions of *TaHMA3, TaHMA2, TaMSRMK3*, and *TraesCS7D02G100200* from the resequencing data of UIP and LCS. The three genes contain 2,487, 2,298, and 1,134 nucleotides and encode 829, 766, and 378 amino acids in coding sequence, respectively. In *TaHMA3* gene sequence of LCS, one SNP (at 1,974 bp G/A) was detected, which resulted in the exchange of amino acids between arginine and glutamine. By predicting the protein structure, the amino acid variation of *TaHMA3* did not change the three-dimensional (3D) structure of protein (Supplementary Figure 1). Four SNPs were detected in *TaHMA2* gene sequence, one SNP (at 3,633 bp A/G) resulted in a synonymous mutation of glycine, and the other three SNPs were non-synonymous mutations (Figure 3). One SNP at 3,094 bp C/A resulted in the exchange of amino acids between leucine and methionine. One SNP at 3,893 bp G/C resulted in the exchange of amino acids between glycine and alanine. The other SNP at 3,963 bp C/G resulted in the exchange of amino acids between isoleucine and methionine. The exchange of a single amino acid at the 338 site of UIP predicted to increase an α-helix on the 3D structure (Supplementary Figure 1). No sequence polymorphism was found in the gene sequences of *TaMSRMK3* and *TraesCS7D02G100200.* The KASP marker for *TaHMA2* shown in Figure 3 was significantly associated with grain Cd content in all environments, except for 17SS (Table 7). The effect of *TaHMA2* was also significantly associated with grain Cd content in 127 diverse spring wheat cultivars and elite lines (Table 8).

**TABLE 7:** Allelic effect of *TaHMA2* on grain Cd content in UI Platinum × LCS Star-derived population.

**Trial**	**Allele**	**Mean Cd**	**Difference^a^**	***P*-value**	**No. of lines**
18SS	UIP	0.0742	−0.0090	0.002	85
	LCS	0.0832			93
18AS	UIP	0.0902	−0.0121	<0.001	85
	LCS	0.1023			93
17SS	UIP	0.1052	−0.0015	0.710	85
	LCS	0.1067			93
17AS	UIP	0.1145	−0.0131	<0.001	85
	LCS	0.1276			93
BLUP	UIP	0.0984	−0.0053	< 0.001	85
	LCS	0.1037			93

*^*a*^The difference is calculated by subtracting the mean of the entries with the LCS allele from the mean of the entries with the UIP allele.*

**TABLE 8:** Allelic effect of *TaHMA2* on grain Cd content in 127 diverse spring wheat lines grown in 17SS.

**QTL/Marker**	**Allele**	**Mean Cd**	**Difference^a^**	***P-*value**	**Sample size**
*Kasp-TaHMA2*	UIP	0.0625	−0.0082	0.008	109
	LCS	0.0707			18

*^*a*^The difference is calculated by subtracting the mean of the entries with the LCS allele from the mean of the entries with the UIP allele.*

## Discussion

### Grain Cadmium Performance and Quantitative Trait Loci Associated With Grain Cadmium Content

The uptake of Cd in plants depends on the plant itself, the concentration of Cd in soil, and the soil properties, such as soil pH, organic matter content, and cation exchange capacity (Eriksson et al., 1996; Benavides et al., 2005; Kim et al., 2016; Zhuang et al., 2021). Soil pH is negatively correlated with Cd content in grain (Kirkham, 2006; Baize et al., 2009). In this study, the soil pH changed from acidic to neutral across the two experimental sites AS and SS. The grain Cd content of the two parents and the population means decreased, although the Cd content in soil was lower in acidic location AS than in the neutral location SS (Table 2). This result supports the conclusion that the soil pH is the most important factor contributing to Cd uptake in wheat (Nan et al., 2002; Liu et al., 2015). In acid soil, Cd is mainly free Cd^2+^, and at neutral or alkaline pH, Cd forms CdCl, CdHCO_3_, and hydrated CdCO_3_, which increases the adsorption capacity of Cd and reduces the accumulation of Cd in plants (Reddy and Patrick, 1977; Sebastian and Prasad, 2014; Volpe et al., 2015; Ismael et al., 2019). Therefore, avoiding soil acidification will reduce the bioavailability of Cd in soil. We also observed a year effect of Cd content in grain. The grain Cd content in parents and in the DHLs in 2018 was lower than in 2017 in the same location. The Cd and Fe content in soil was higher in 2018 than in 2017, which might be the cause of the year effect.

Three QTL regions, namely, *QCd.uia2-5B*, *QCd.uia2-7B*, and *QCd.uia2-7D*, were identified in 2–3 location-year trials. *QCd.uia2-7B* and *QCd.uia2-7D* are novel QTL in common wheat. The three QTL regions have additive effects that can be used in breeding low grain Cd cultivars. However, none of QTL regions was detected in all four trials. *QCd.uia2-5B* was only detected in the two 2018 trials (18SS and 18AS), *QCd.uia2-7B* only in AS trials 17AS and 18AS, and *QCd.uia2-7D* was detected in three of the four trials. The effects of the three QTL regions were generally small, explaining up to 12% of total phenotypic variation (Table 4). The Cd content of grain in common wheat is generally much lower than that in durum and rice, and the two parents did not differ greatly in grain Cd content, which possibly explains the small effect of the three QTL identified in this study. To improve the power of QTL detection for grain Cd content, it is essential to do Cd screening of grain in controlled environments and using near-isogenic lines.

### Candidate Genes in the Intervals of the Three Quantitative Trait Loci for Grain Cadmium Content

Based on the physical location, annotation of candidate gene function, and comparison of homologous genes, we found three genes that regulate the uptake and transport of Cd in durum and/or rice and also identified three orthologous genes in wheat, namely, *TaHMA3* (*TraesCS 5B02G388000*), *TaHMA2* (*TraesCS7B02G320900*), and *TaMSRMK3* (*TraesCS7B02G322 900*). *TaHMA3* encodes a P_1B_-type heavy metal ATPase 3 (HMA3) that is orthologous to *OsHMA3.* OsHMA3 is a transporter protein located on the vacuolar membrane of the root, which can transport Cd absorbed by root to the vacuole, thus limiting the transport of Cd to the aboveground plant parts (Miyadate et al., 2011; Sasaki et al., 2014; Maccaferri et al., 2019; Lei et al., 2020). *TaHMA2* is orthologous to rice *HMA2* (*OsHMA2*). OsHMA2, a type of efflux metal transporter expressed on the cell membrane, is involved in root-to-shoot transport and plays a role in Zn and Cd loading into the xylem (Satoh-Nagasawa et al., 2012; Takahashi et al., 2012). *TaMSRMK3* is orthologous to rice *OsMSRMK3*. The expression of OsMSRMK3 is upregulated by heavy metal stress (Agrawal et al., 2003).

Based on the sequence comparison of the three candidate genes, *TaHMA2* was a candidate gene validated using the gene-specific KASP marker. The function of *TaHMA2* is being sought *via* gene-editing technology in an ongoing project. This finding is an important starting point for understanding the molecular mechanism of Cd absorption, transport, and accumulation in wheat and provides a theoretical basis for breeding low cadmium varieties using molecular technology.

## Data Availability Statement

The datasets presented in this study can be found in online repositories. The names of the repository/repositories and accession number(s) can be found in the article/Supplementary Material.

## Author Contributions

JC and LQ designed the experiment and developed the original manuscript. LQ, JZe, MS, and JZa did sequence analysis and genotyping of the candidate genes. JC, LQ, JW, RW, KI, NK, and WZ did the field experiments. LQ, RW, and JZa performed the phenotypic data analysis and QTL detection. JC, LQ, JZe, and JZa revised the manuscript. All authors approved the submitted version of the manuscript.

## Conflict of Interest

The authors declare that the research was conducted in the absence of any commercial or financial relationships that could be construed as a potential conflict of interest.

## Publisher’s Note

All claims expressed in this article are solely those of the authors and do not necessarily represent those of their affiliated organizations, or those of the publisher, the editors and the reviewers. Any product that may be evaluated in this article, or claim that may be made by its manufacturer, is not guaranteed or endorsed by the publisher.

## Footnotes

1
http://usual.usu.edu/

2
http://www.jmp.com/

3
http://wheat.pw.usda.gov/ggpages/wgc/98/Intro.htm

4
https://urgi.versailles.inra.fr/blast/?dbgroup=wheat_iwgsc_refseq_v1_chromosomes

5
http://202.194.139.32

6
http://rice.plantbiology.msu.edu/

7
http://plants.ensembl.org/index.html

8
https://github.com/tanghaibao/jcvi/wiki/MCscan-(Python-version)
