# Supplementary material for: Novel Quantitative Trait Loci for Grain Cadmium Content Identified in Hard White Spring Wheat
Source: Front Plant Sci. 2021 Dec 2;12:756741. doi: 10.3389/fpls.2021.756741 (PMC8678907; doi:10.3389/fpls.2021.756741)
Supplement: Supplementary file 1 [file Data_Sheet_1.docx]

**Supplementary Tables**

**Table S1.** Epistatic QTLs analysis for Cd content with the EPI module

| Traits | eQTL1 | Position1 | Left  Marker1 | Right  Marker1 | eQTL2 | Position2 | Left  Marker2 | Right  Marker2 | LOD | PVE  (%) | Add1 | Add2 | AddbyAdd |
| --- | --- | --- | --- | --- | --- | --- | --- | --- | --- | --- | --- | --- | --- |
| 18SSCd | *QCd.uia2-2D.2* | 57 | IWB12136 | IWB48312 | *QCd.uia2-5B.2* | 58 | IWB60833 | IWB44014 | 5.26 | 6.60 | 8.17 | -2.97 | -63.48 |
| 18SSCd | *QCd.uia2-2B.1* | 213 | IWB25553 | IWB48218 | *QCd.uia2-3B* | 212 | IWB14318 | IWB23457 | 5.29 | 9.64 | 16.66 | -18.93 | -84.84 |
| 18ASCd | *QCd.uia2-7A.1* | 22 | IWB8722 | IWB28062 | *QCd.uia2-6B* | 10 | IWB48362 | IWB27812 | 5.27 | 5.61 | -4.81 | 15.03 | 39.31 |
| 17ASCd | *QCd.uia2-1D* | 111 | IWB56444 | IWB6677 | *QCd.uia2-7A.2* | 33 | IWB35860 | IWB10036 | 5.54 | 9.93 | -9.49 | -12.88 | -66.63 |
| BLUP | *QCd.uia2-2D.3* | 174 | IWB11313 | IWB9546 | *QCd.uia2-3D* | 0 | IWB50723 | IWB16145 | 5.31 | 8.27 | 2.32 | 37.46 | -39.70 |
| BLUP | *QCd.uia2-2B.2* | 102 | IWB1188 | IWB79793 | *QCd.uia2-4B.2* | 212 | IWB14318 | IWB23457 | 5.99 | 8.78 | -4.11 | 17.55 | -30.15 |

PVE is short for phenotypic variation explained; Add represents the additive effect (*a*); AddbyAdd represents the epistatic effect (*aa*)

**Supplementary Figures**

**Figure. S1** Predicted 3D structural model of *TaHMA3* and *TaHMA2* in UI Platinum and LCS Star.
